# Photoreceptor degeneration in microphthalmia (*Mitf*) mice: partial rescue by pigment epithelium-derived factor

**DOI:** 10.1242/dmm.035642

**Published:** 2019-01-11

**Authors:** Yu Chen, Juan Yang, Huiqin Geng, Liping Li, Jinyang Li, Bing Cheng, Xiaoyin Ma, Huirong Li, Ling Hou

**Affiliations:** Laboratory of Developmental Cell Biology and Disease, School of Ophthalmology and Optometry and Eye Hospital, State Key Laboratory of Optometry, Ophthalmology, and Vision Science and Zhejiang Provincial Key Laboratory of Ophthalmology, Wenzhou Medical University, Wenzhou 325003, China

**Keywords:** RPE, PEDF, MITF, Neurodegeneration, Retina

## Abstract

Dysfunction and loss of the retinal pigment epithelium (RPE) are hallmarks of retinal degeneration, but the underlying pathogenetic processes are only partially understood. Using mice with a null mutation in the transcription factor gene *Mitf*, in which RPE deficiencies are associated with retinal degeneration, we evaluated the role of trophic factors secreted by the RPE in retinal homeostasis. In such mice, the thickness of the outer nuclear layer (ONL) is as in wild type up to postnatal day 10, but then is progressively reduced, associated with a marked increase in the number of apoptotic cells and a decline in staining for rhodopsin. We show that retinal degeneration and decrease in rhodopsin staining can be prevented partially in three different ways: first, by recombining mutant-derived postnatal retina with postnatal wild-type RPE in tissue explant cultures; second, by adding to cultured mutant retina the trophic factor pigment epithelium-derived factor (PEDF; also known as SERPINF1), which is normally produced in RPE under the control of *Mitf*; and third, by treating the eyes of *Mitf* mutant mice *in vivo* with drops containing a bioactive PEDF 17-mer peptide. This latter treatment also led to marked increases in a number of rod and cone genes. The results indicate that RPE-derived trophic factors, in particular PEDF, are instrumental in retinal homeostasis, and suggest that PEDF or its bioactive fragments may have therapeutic potential in RPE deficiency-associated retinal degeneration.

## Abstract (precis)

**Summary:** This study reveals that the MITF-PEDF pathway plays a critical role in RPE homeostasis and that simple eye drops of a PEDF 17-mer peptide can partially prevent *Mitf* deficiency-associated retinal degeneration in mice.

## INTRODUCTION

Retinal degenerations are a group of clinically and genetically heterogeneous disorders characterized by progressive loss of vision due to degeneration of photoreceptors. Retinal degenerative diseases, which include age-related macular degeneration (AMD), cone dystrophy and retinitis pigmentosa, are the leading causes of blindness in the developed world (Bramall et al., 2010; Hartong et al., 2006; Lim et al., 2012). Among them, AMD is the major cause of vision loss in older adults and its pathogenesis is initiated by dysfunction, degeneration and loss of retinal pigment epithelium (RPE) cells. These cells play important roles during eye development, retinal homeostasis and visual function (Strauss, 2005). They absorb scattered light, secrete neurotrophic factors, maintain the blood-retinal barrier, phagocytose detached photoreceptor outer segments, participate in the visual cycle and provide antioxidant functions (Strauss, 2005). Not surprisingly, then, defects or dysfunction in RPE cells often lead to photoreceptor dysfunction, retinal degeneration and blindness (Ben M'Barek et al., 2017; Kolomeyer and Zarbin, 2014; Longbottom et al., 2009; Mullen and LaVail, 1976; Raymond and Jackson, 1995; Strauss, 2005).

RPE cells secrete a variety of neurotrophic factors including pigment epithelium-derived factor [PEDF (also known as SERPINF1), a 50 kDa member of the serpin superfamily (Steele et al., 1993)], brain-derived neurotrophic factor (BDNF), nerve growth factor (NGF), and platelet-derived growth factor (PDGF), transforming growth factor-β (TGF-β) and vascular endothelial growth factor (VEGF) (Bharti et al., 2006; Kolomeyer and Zarbin, 2014; Strauss, 2005) proteins. All of these factors support the survival of photoreceptors and maintain the integrity of the retina (Barnstable and Tombran-Tink, 2004; Dawson et al., 1999; Kolomeyer and Zarbin, 2014; Takahashi et al., 2017; Wang et al., 2013, 2014), and all might potentially be used to treat photoreceptor degeneration in humans (Kolomeyer and Zarbin, 2014). PEDF is a multifunctional factor with neurotrophic, neuroprotective and antiangiogenic activities (Barnstable and Tombran-Tink, 2004; Becerra, 2006; Becerra et al., 2004; Tombran-Tink and Barnstable, 2003), and disturbances in its secretion play important roles in the pathophysiology of retinal degenerations, including AMD and retinitis pigmentosa (Chaum, 2003)*.* In fact, PEDF expression levels are significantly decreased in patients with AMD and other neuroretinal dystrophic diseases (Bhutto et al., 2006; Holekamp et al., 2002; Kolomeyer et al., 2011; Ogata et al., 2004). How these neurotrophic factors are regulated in health and disease, however, is still incompletely understood.

Microphthalmia-associated transcription factor (MITF) is a member of the MITF-TFE family of basic-helix-loop-helix-leucine zipper (bHLHZip) transcription factors that is expressed in a variety of cell types, notably melanin-bearing pigment cells including RPE cells, in which it plays important roles in their development and function (Arnheiter, 2010; Bharti et al., 2006; Hou and Pavan, 2008). In humans, heterozygous mutations in *MITF* are associated with Waardenburg syndrome type 2 and Tietz syndrome, which are characterized by deafness, and which can include premature hair graying and heterochromia iridis (Amiel et al., 1998; Tassabehji et al., 1994). Biallelic MITF mutations may lead to the recently recognized COMMAD syndrome, which is characterized by the combination of coloboma, osteopetrosis, microphthalmia, macrocephaly, albinism and deafness (George et al., 2016). These phenotypes reflect what is seen in mice homozygous for severe mutant alleles of *Mitf*. As *Mitf* is expressed prominently in the RPE, its mutations lead to aberrant RPE development (Bharti et al., 2008; Bumsted and Barnstable, 2000; Hodgkinson et al., 1993; Nguyen and Arnheiter, 2000; Steingrímsson et al., 1996; Tsukiji et al., 2009) and to the development of an abnormally small eye (microphthalmia) associated with retinal degeneration (Möller et al., 2004; Smith, 1992; Smith and Hamasaki, 1994; Steingrímsson et al., 1996). We have previously shown that MITF regulates the expression of neurotrophic factors including PEDF in RPE cells (Ma et al., 2012), but whether *Mitf*-regulated neurotrophins play any role in retinal degeneration remains unknown.

In the present study, we specifically address the question of the role of PEDF in *Mitf* deficiency-associated retinal degeneration because we found *Pedf* expression to be significantly decreased in *Mitf*-deficient RPE. Interestingly, in an explant culture system, the addition of wild-type (WT) RPE to retina from *Mitf*-deficient eyes partially rescued the degeneration of photoreceptors and retinal thickness. Moreover, partial rescue could also be achieved by addition of exogenous PEDF or a PEDF peptide 17-mer (PEDF 17-mer) in explant cultures, and, remarkably, application of eye drops containing the PEDF 17-mer to the eyes of *Mitf*-deficient mice. Hence, it appears that the MITF-PEDF pathway in RPE cells is an important contributor to photoreceptor health and retinal homeostasis.

## RESULTS

### Progressive degeneration in *Mitf*-deficient retina

**Fig. 1.**
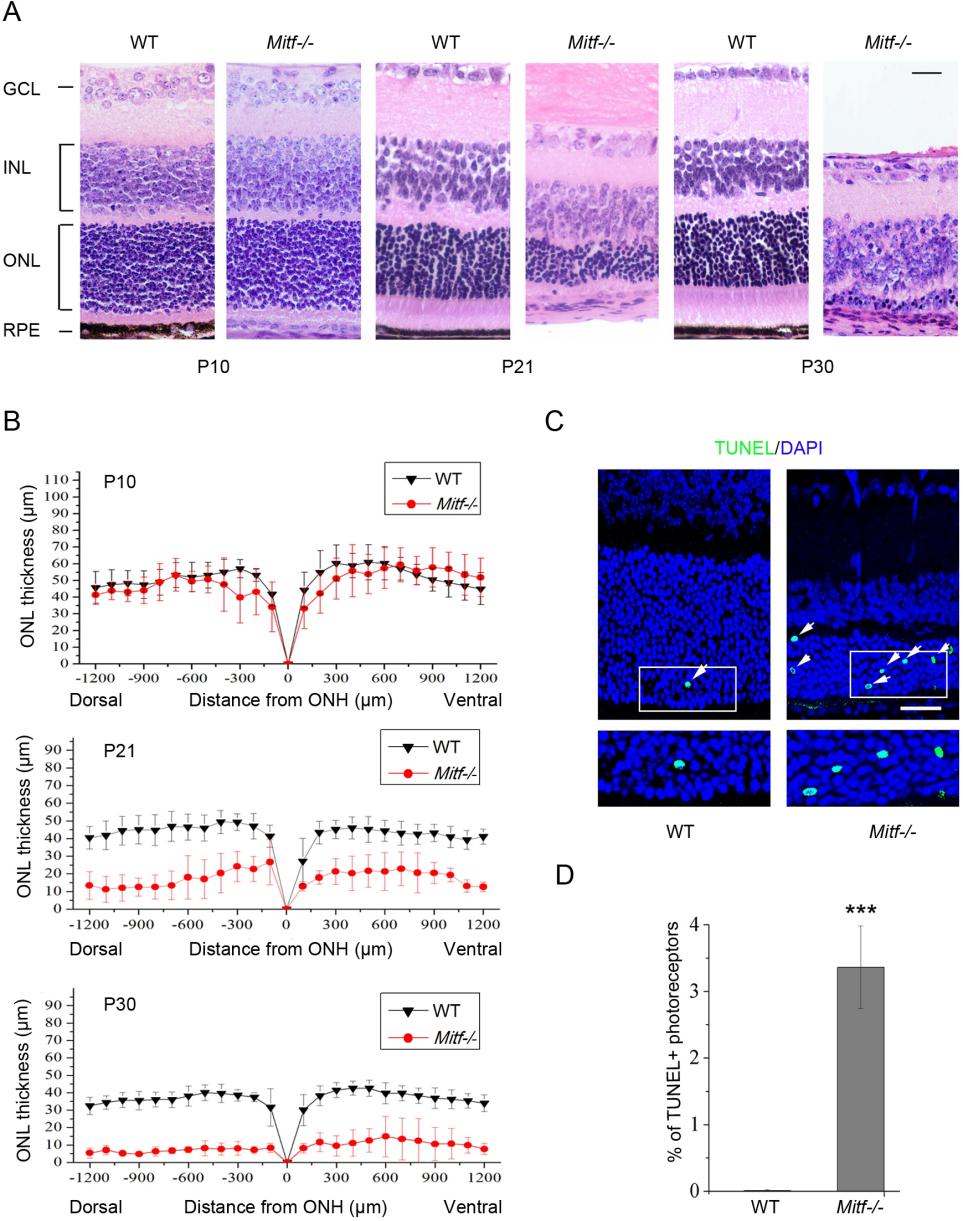
**Progressive photoreceptor cell degeneration in *Mitf^−/−^* retina.** (A) Representative examples of Hematoxylin-Eosin staining of paraffin-embedded sections of wild-type (WT) and *Mitf^−/−^* retinas at P10, P21 and P30. Note that *Mitf^−/−^* eyes lack pigmentation in the RPE layer and from P21 to P30 show a progressive reduction in retinal thickness, particularly in the ONL. GCL, ganglion cell layer; INL, inner nuclear layer; ONL, photoreceptor outer nuclear layer; RPE, retinal pigment epithelium. Scale bar: 20 μm. (B) Quantification of the ONL thickness from the optic nerve head (ONH) to the peripheral retina for WT (*n*=8) or *Mitf^−/−^* mice (*n*=6). Note that from P21 to P30, the ONL thickness in retinas of *Mitf^−/−^* mice was significantly decreased compared with that in corresponding retinas of WT mice. (C) Representative images for TUNEL staining (green) of WT and *Mitf^−/−^* retinas at P21. The arrows and white line boxes (including the enlarged insets) mark TUNEL-positive photoreceptor cells. Nuclei were stained with 4′,6-diamidino-2-phenylindole (DAPI, blue). Scale bar: 20 μm. (D) Quantification of the TUNEL-positive cells was performed based on the results from C. Note that the percentage of TUNEL-positive photoreceptor cells/total photoreceptor cells was significantly increased in *Mitf^−/−^* compared with WT retina. Results are presented as mean±s.d. ***P<0.001.

It is well known that mice homozygous for the *Mitf* null allele *Mitf^mi-^^vga9^* (hereafter referred to as *Mitf^−/−^* mice) show abnormal eye development and postnatal retinal degeneration (Hodgkinson et al., 1993; Nguyen and Arnheiter, 2000), as do mice homozygous for the original *Mitf^mi^* mutation (Bumsted and Barnstable, 2000). We here analyzed the changes in retinal morphology and integrity at different postnatal stages of *Mitf^−/−^* retinas. At postnatal day (P) 10, the thickness of the outer nuclear layer (ONL) was similar in WT and *Mitf^−/−^* retinas but the *Mitf^−/−^* RPE lacked pigmentation (Fig. 1A). From P21 to P30, however, the ONL of *Mitf^−/−^* mice became progressively thinner (Fig. 1A,B). This loss of tissue was likely due to increased photoreceptor cell death, as indicated by an increase in TUNEL-positive, apoptotic cells (Fig. 1C,D). Apoptotic photoreceptor cells were also marked by cleaved caspase-3 (Fig. S1A,B), suggesting that the cells become apoptotic by a caspase-3-dependent pathway. This interpretation is supported by the absence of nuclear translocation of apoptosis-inducing factor (AIF; also known as AIFM1) (Fig. S1C,D); the translocation of AIF from mitochondria to nuclei is a hallmark of the caspase-independent pathway of apoptosis (Krantic et al., 2007), and AIF is the main mediator of the caspase-independent apoptosis in *rd1* mice and RCS rats (Mizukoshi et al., 2010; Sanges et al., 2006). These data indicate that photoreceptor cells progressively degenerate in *Mitf^−/−^* mice, suggesting that MITF plays a protective role in photoreceptor cells.

### WT RPE cells partially rescue degeneration of *Mitf^−/−^* retinas

**Fig. 2.**
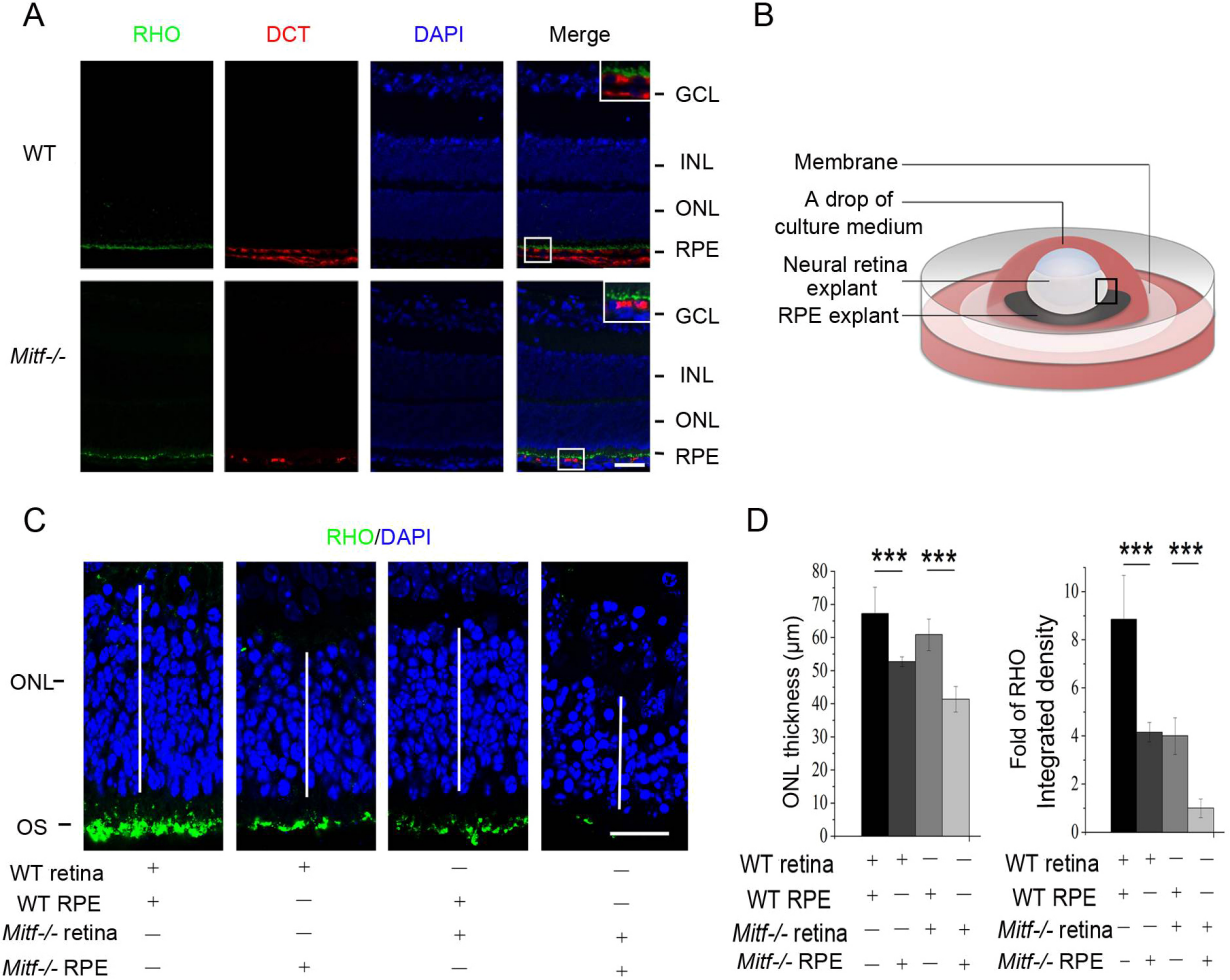
**WT** **RPE partially rescues *Mitf*^−/−^ photoreceptors and integrity of *Mitf^−/−^* retina.** (A) Double immunostaining for photoreceptors (RHO, green) and the pigment enzyme dopachrome tautomerase (DCT, red) was performed on WT and *Mitf^−/−^* retinas at P10 [nuclei were stained with DAPI (blue)]. The white line boxes on the right are enlarged areas containing DCT-positive RPE cells. Note that the double layer of red cells in WT is due to the presence of both DCT-positive RPE and choroidal cells, while in mutant, only DCT-positive RPE cells remain. GCL, ganglion cell layer; INL, inner nuclear layer; ONL, photoreceptor outer nuclear layer; RPE, retinal pigment epithelium. Scale bar: 20 μm. (B) Schematic representation of neural retina and RPE explant experiments. In a first step, RPE-choroid-sclera complex (RPE explant) or neural retina explants were isolated from WT or *Mitf^−/−^* mice at P8 and then recombined as indicated in C. The black line box indicates the area of the retina that was analyzed. (C) Ten days after recombination, neural retina was analyzed by immunostaining for RHO (green). White lines mark the thickness of the ONL. OS, outer segment. Scale bar: 20 μm. (D) Quantification of ONL thickness and the integrated density of RHO was performed based on the results from C. Note that when WT neural retina was recombined with *Mitf^−/−^* RPE, the RHO staining was lower and the ONL was thinner than when WT RPE was used. In *Mitf^−/−^* retina, RHO staining and ONL thickness was markedly improved when WT RPE was used for recombination, but not when *Mitf^−/−^* RPE was used. Results are presented as mean±s.d. ***P<0.001.

The above experiments showing an association of RPE abnormalities with photoreceptor degeneration do not formally prove a causal relationship, as one isoform of MITF, A-MITF, is also expressed in the retina throughout development, although at low levels (Bharti et al., 2008). We, therefore, asked whether addition of a WT RPE to *Mitf^−/−^* retina might rescue photoreceptor loss. To this end, we used explant cultures allowing us to deliberately recombine WT RPEs with *Mitf^−/−^* retinas and vice versa. These experiments were feasible because, as shown in Fig. 2A, dopachrome tautomerase (DCT) antibody-positive RPE cells were still detected in P10 *Mitf^−/−^* eyes, suggesting that such cells existed at least up to this time point. Hence, we chose to prepare explants from P8 mice and separated them into RPE and retina/lens as described in the Materials and Methods and in the Fig. 2 legend. We then recombined retinas and RPEs with the different genotypes as indicated in Fig. 2B and C. After a culture period of 10 days, we analyzed a retina section that was not in direct physical contact with the RPE for thickness and photoreceptor outer segment staining (Fig. 2B). As shown in Fig. 2C and D, the reconstitution of WT retina with WT RPE led to normal rhodopsin (RHO) expression and normal retinal thickness, whereas the reconstitution of *Mitf^−/−^* retina with *Mitf^−/−^* RPE showed reduced rhodopsin expression and much decreased ONL thickness. As expected, when WT retinas were reconstituted with *Mitf^−/−^* RPE, rhodopsin expression and ONL thickness were reduced. In contrast, when *Mitf^−/−^* retina was reconstituted with WT RPE, rhodopsin expression and ONL thickness were partially restored. These results suggest that the WT RPE produces a trophic factor or factors that are able to overcome the process of retinal degeneration seen in *Mitf^−/−^* eyes.

### Reduced expression of growth factors in *Mitf^−/−^* RPE *in vivo*

**Fig. 3.**
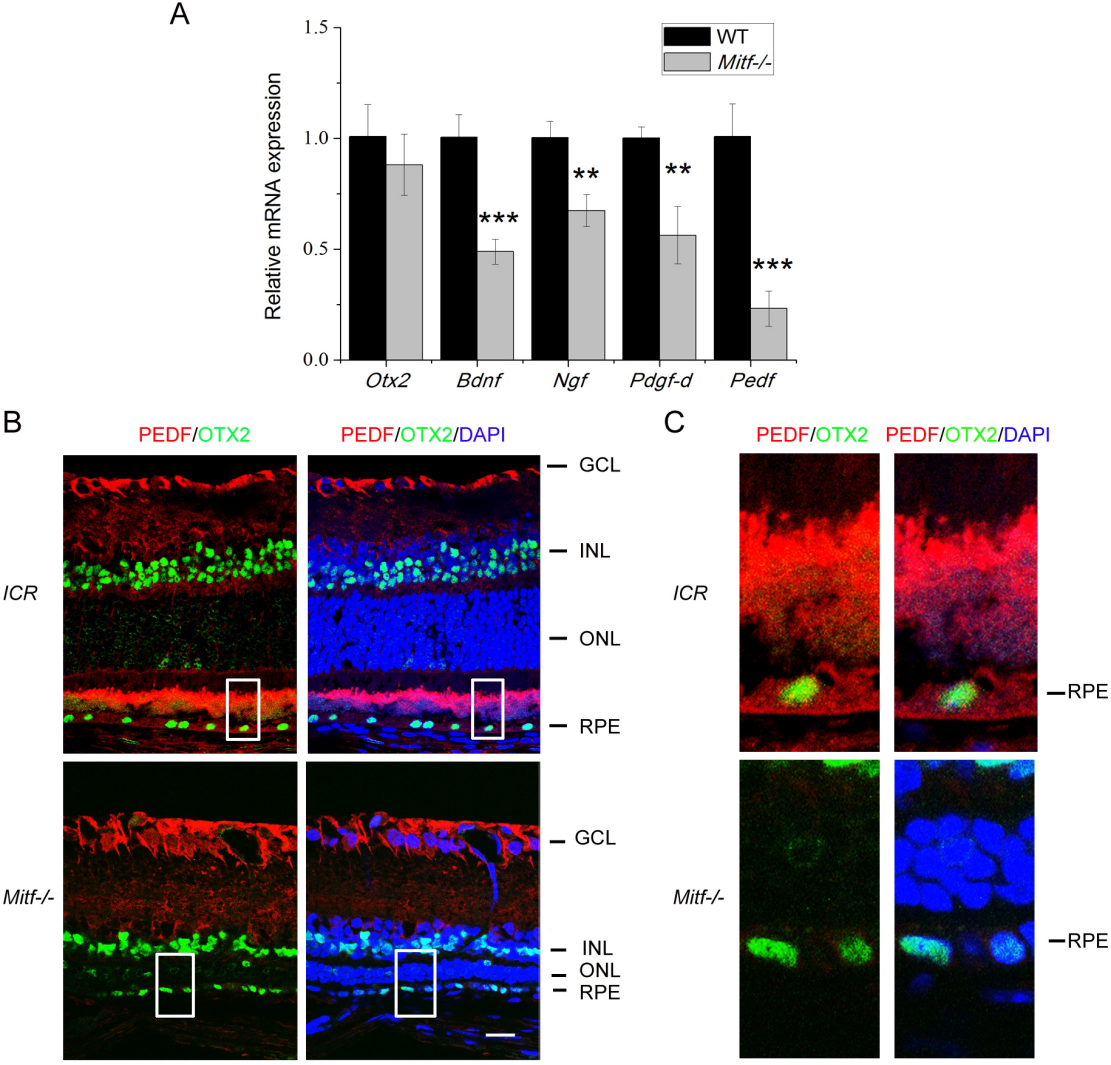
**Downregulation of *Pedf* in *Mitf^−/−^* RPE cells.** (A) Relative RNA expression levels of the indicated neurotrophic factor genes were evaluated in RPE cells of P21 WT or *Mitf^−/−^* mice by real-time PCR. *n*=4. Results are presented as mean±s.d. **P<0.01, ***P<0.001. (B) Double immunostaining for OTX2 (which labels RPE) and PEDF was performed on WT retina (ICR strain) or *Mitf^−/−^* retina at P21 [nuclei were stained with DAPI (blue)]. GCL, ganglion cell layer; INL, inner nuclear layer; ONL, photoreceptor outer nuclear layer; RPE, retinal pigment epithelium. Scale bar: 20 μm. (C) Enlarged images of the white line boxes in B. Note that PEDF staining can be detected in ICR RPE, interphotoreceptor matrix (IPM) area and retinal GCL, but is undetectable in *Mitf^−/−^* RPE and IPM, although still expressed in *Mitf^−/−^* GCL.

Previous evidence indicated that RPEs indeed produce a number of trophic factors supporting retinal maintenance (reviewed in Bharti et al., 2006; Strauss, 2005), and we have shown that *Mitf* regulates the expression of some of these factors in RPE cells *in vitro* (Ma et al., 2012). We thus analyzed whether *Mitf* also regulates the expression of neurotrophic factors in RPE cells *in vivo*. To this end, we analyzed the expression levels of several RPE-derived neurotrophic factors in freshly isolated RPE from WT and *Mitf^−/−^* mice. As shown in Fig. 3, mRNA expression levels of *Bdnf*, *Ngf*, *Pdgf-d* and *Pedf* were significantly decreased in RPE from *Mitf^−/−^* mice at P21, as measured by real-time PCR (Fig. 3A). Among them, PEDF was of particular interest because it is known to have neurotrophic, neuroprotective and antiangiogenic activities (Becerra, 2006; Tombran-Tink and Barnstable, 2003), and to be regulated directly by MITF (Fernández-Barral et al., 2014; Dadras et al., 2015). Immunostaining analysis confirmed that PEDF was present in RPE of WT ICR mice (used because they lack melanin, which interferes with immunofluorescence signals), but was hardly detectable in *Mitf^−/−^* RPE cells (which, as mentioned, also lack melanin), as characterized by OTX2 expression (Fig. 3B,C). These results suggest that the loss of functional MITF leads to a reduction in PEDF expression, and that defects in endogenous PEDF production in the *Mitf^−/−^* RPE may contribute to the *Mitf* deficiency-associated retinal degeneration.

### PEDF treatment partially rescues *Mitf^−/−^* retinal degeneration

**Fig. 4.**
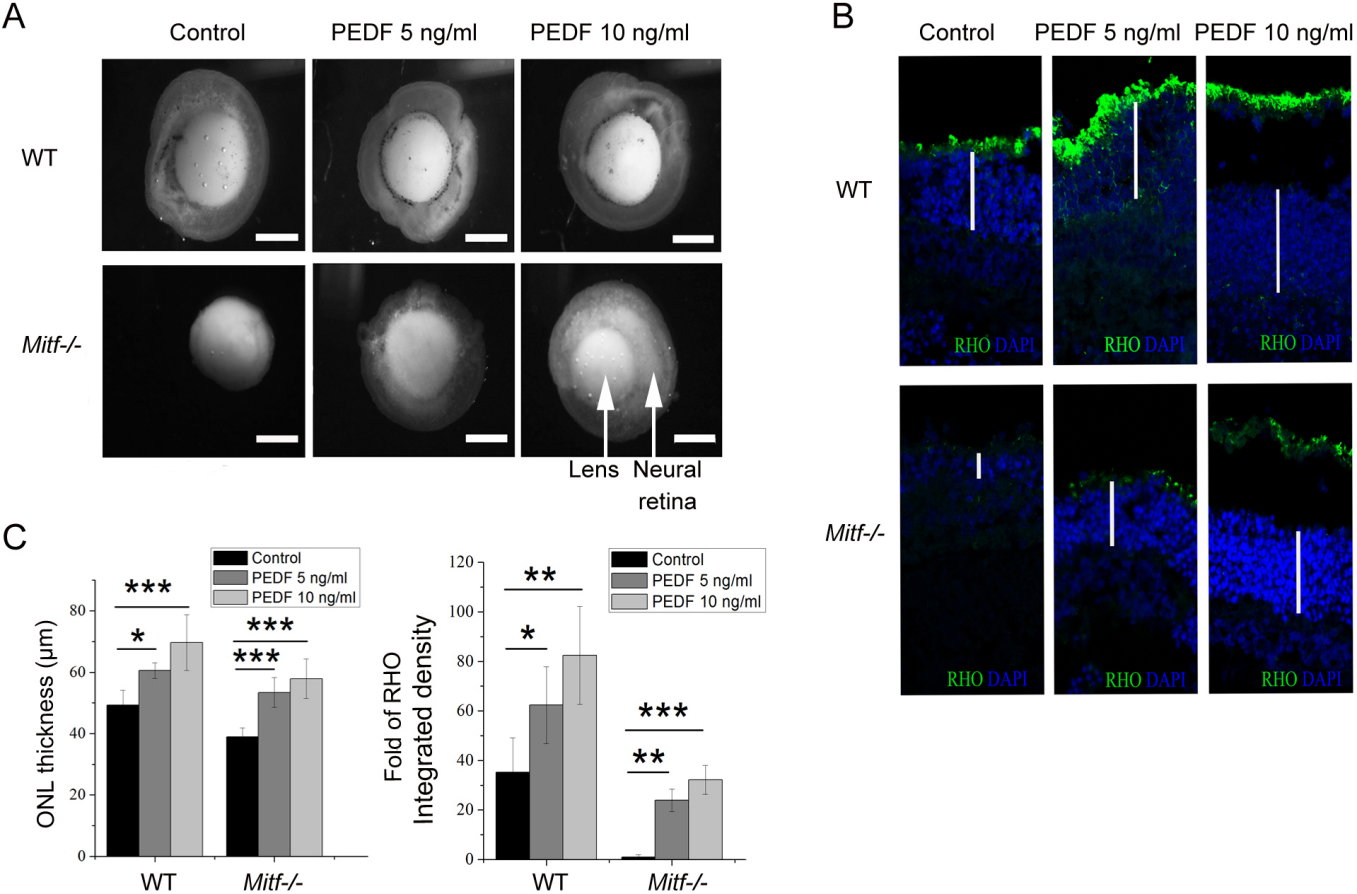
**PEDF rescues *Mitf^−/−^* retinal degeneration in a dose-dependent** **manner** **in explant cultures.** (A) Neural retina explants of WT or *Mitf^−/−^* mice were prepared and treated with PEDF at the indicated doses (representative examples of *n*=5). Scale bars: 100 μm. (B) RHO immunostaining in representative sections of neural retina explants. White lines mark the thickness of the outer nuclear layer (ONL). (C) Quantification of ONL thickness and the integrated density of RHO was performed based on the results from B (*n*=5). Note that in *Mitf^−/−^* retina, both the thickness of the ONL and RHO staining increased with increasing PEDF dose. Results are presented as mean±s.d. *P<0.05, **P<0.01, ***P<0.001.

To substantiate the contribution of PEDF to retinal homeostasis, we first cultured *Mitf^−/−^* neural retina explants in the absence or presence of two different doses of exogenous PEDF as indicated in Fig. 4. In fact, addition of PEDF partially restored expression of rhodopsin and the thickness of the ONL in a dose-dependent manner. These results suggest that PEDF can rescue *Mitf^−/−^* retina degeneration, at least in explant cultures.

**Fig. 5.**
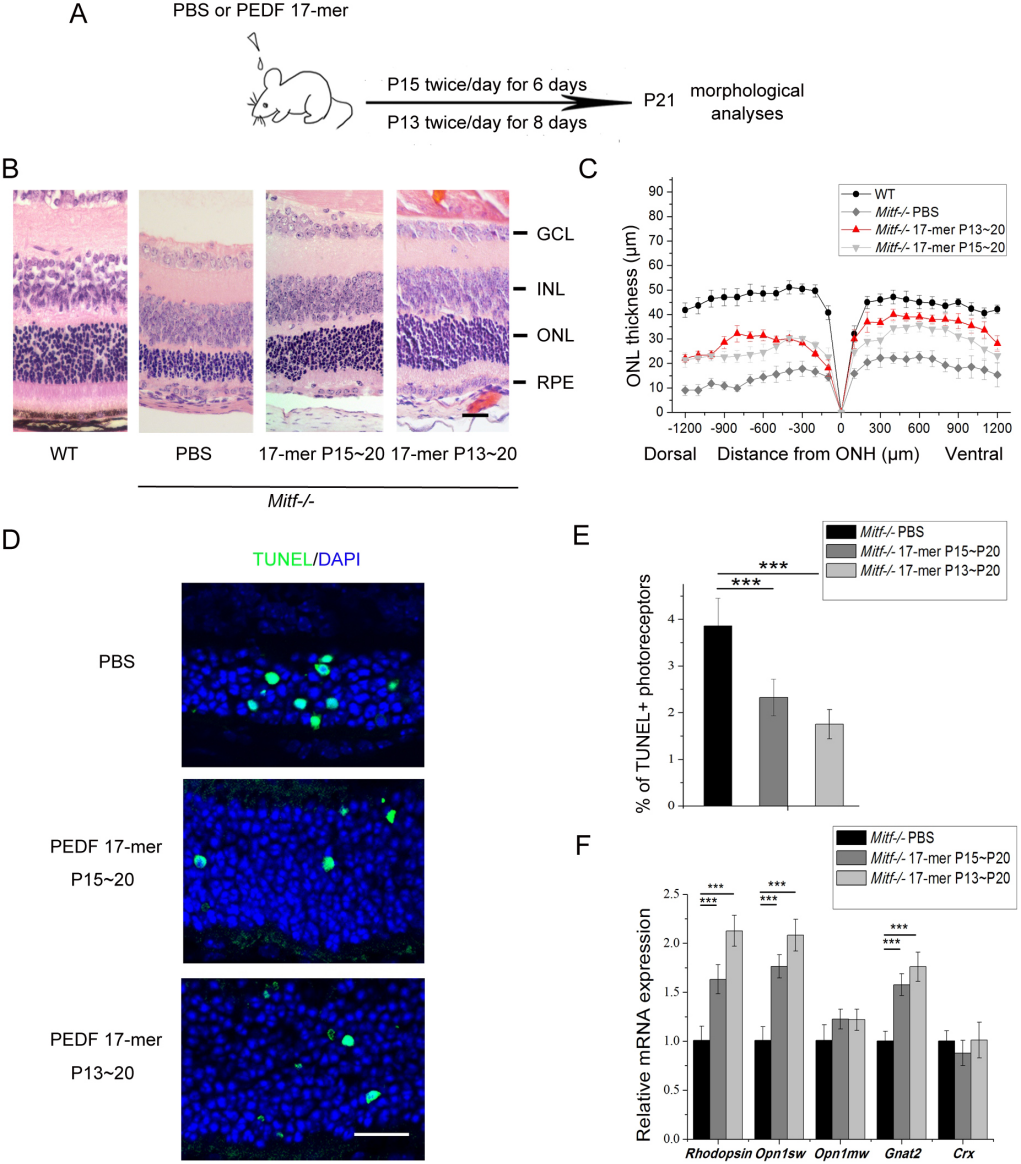
**Treatment with the PEDF 17-mer peptide partially rescues *Mitf^−/−^* retinal degeneration *in vivo*.** (A) Schematic representation of PEDF 17-mer peptide treatment in *Mitf^−/−^* eye. Drops of PBS or PBS containing the 17-mer at 1 mg/ml were applied topically twice/day from P15 to P20 or from P13 to P20. Eyes were harvested and morphologically analyzed at P21. (B,C) Histological analysis (B) and quantification of the ONL thickness from the optic nerve head (ONH) to the peripheral retina (C) of untreated WT eyes or *Mitf^−/−^* eyes after treatment with PBS (*n*=6) or PBS+peptide (*n*=6). Note that the ONL thickness was increased in the peptide-treated *Mitf^−/−^* eye. The delivery of PEDF 17-mer at P13 results in an improvement over delivery at P15. Scale bar: 20 μm. (D) TUNEL staining (green) in *Mitf^−/−^* control and peptide-treated eyes (*n*=5) at P21 [nuclei were stained with DAPI (blue)]. Scale bar: 20 μm. (E) The percentage of TUNEL-positive cells/total photoreceptor cells was determined from D. Note the decrease after PEDF 17-mer peptide treatment. (F) RNA expression levels of several photoreceptor-associated genes in peptide-treated eyes relative to those in control-treated eyes as evaluated by real-time PCR. Note that the expression levels of *Rho*, *Opn1sw* and *Gnat2* were partially restored after treatment. Results are presented as mean±s.d. ***P<0.001.

We then tested whether PEDF might also have a similar rescue effect *in vivo*. Therapeutically, PEDF is usually injected intravitreally, but as intravitreal injection can lead to eye damage, we sought to apply PEDF externally. It has been shown previously that a small peptide fragment, PEDF 17-mer (positions 98-114) from the neurotrophic region of PEDF retains key interacting residues for binding PEDF receptor and shares with full-length PEDF substantial retina-protective properties (Kenealey et al., 2015). Hence, we first cultured *Mitf^−/−^* neural retina explants in the absence or presence of different doses of the exogenous PEDF 17-mer as indicated in Fig. S2. Addition of the PEDF 17-mer partially restored the expression of rhodopsin and the thickness of *Mitf^−/−^* ONL in a dose-dependent manner (Fig. S2). Based on this result, we applied the PEDF 17-mer at 1 mg/ml to eyes of mice from P13 to P20, or from P15 to P20, twice daily, using eye drops, and examined them at P21 (Fig. 5A). As shown in Fig. 5B and C, after application of PBS solvent lacking PEDF 17-mer, *Mitf^−/−^* retinas underwent severe retinal degeneration. In contrast, when treated with the PEDF 17-mer, they showed a markedly better retinal structure, whereby application of PEDF 17-mer at P13 led to a more pronounced rescue compared with application at P15. It is not clear, however, whether this difference is due to the differences in time point, or differences in duration, of peptide application. Nevertheless, in both conditions, rescue was marked by a significant reduction in TUNEL^+^ apoptotic cells (Fig. 5D,E).

Lastly, we tested whether the partial rescue of retinal structure and the reduction in the number of apoptotic cells was paralleled by increased expression of rod and cone genes, which is normally lost during retinal degeneration (Tang et al., 2010). Indeed, as shown in Fig. 5F, the expression of the rod-related genes *Rho* and *Crx*, and of the cone-related genes *Opn1sw*, *Opn1mw*, *Gnat2* and *Crx*, was increased after treatment with the PEDF 17-mer peptide. Taken together, these results indicate that application of the PEDF 17-mer peptide enhanced photoreceptor survival and partially prevented retinal degeneration in *Mitf* mutant mice, not only *in vitro* but also *in vivo*.

## DISCUSSION

RPE cell functions are controlled by transcription factors and signaling pathways and are critical for the maintenance of retinal anatomy and function. Here, we show that the transcription factor MITF, by regulating the expression of PEDF, plays an important role in normal retinal homeostasis. This conclusion is based on the facts that, as previously shown, MITF regulates the expression of PEDF in human RPE cells in culture, that PEDF expression is lacking in *Mitf*-deficient mouse eyes, and that photoreceptor degeneration in *Mitf*-deficient retinas can be partially rescued *in vitro* by co-culture with WT RPE or by addition of PEDF or a PEDF peptide fragment, and also *in vivo* by the addition of the same fragment. These results suggest that WT RPE cells maintain retinal structure and gene expression in *Mitf^−/−^* mutant mice by secreting PEDF, although it is likely that other neurotrophic factors are also involved. This is consistent with previous studies showing that PEDF could delay photoreceptor degeneration by inhibiting apoptosis (Akiyama et al., 2012; Imai et al., 2005; Wang et al., 2013).

Nevertheless, the PEDF-mediated rescue of retina structure and gene expression in *Mitf*-deficient mouse eyes was only partial in *Mitf*^−/−^ mutant mice. This was not surprising as only the combinations of PEDF with other factors can lead to full rescue (Hua et al., 2018; Wen et al., 2016). Also, it is conceivable that absence of the low levels of MITF normally found in the developing retina might have contributed in a PEDF-resistant way to retinal damage in *Mitf^−/−^* mice. Further studies including RPE-targeted conditional disruption of *Mitf* in the RPE would be needed to obtain a more complete picture of the specific contribution of the RPE to retinal degeneration.

It is well known that PEDF is deficient in patients with AMD (Bhutto et al., 2006; Holekamp et al., 2002; Kolomeyer et al., 2011) and other retinal dystrophic diseases (Ogata et al., 2004), suggesting that PEDF deficiencies contribute to retinal degenerations. Administration of PEDF in models of retinal degeneration, such as *rd1* and *rds* mice or RCS rats (Cayouette et al., 1999; Comitato et al., 2018; Miyazaki et al., 2003; Wang et al., 2013), or in models of light-induced retinal damage (Cao et al., 2001; Imai et al., 2005), can delay photoreceptor degeneration. PEDF can be delivered by intravitreal injection (Cayouette et al., 1999; Comitato et al., 2018; Wang et al., 2013), simian lentivirus (SIV)-mediated gene transfer (Miyazaki et al., 2003; Murakami et al., 2008), adenoviral vectors (Imai et al., 2005) or as nanoparticles (PEDF-NPs) (Akiyama et al., 2012). In order to avoid side effects of intravitreal injections or application of viral vectors, we applied a bioactive PEDF fragment in eye drops as done for treatments in a diabetic retinopathy mouse model (Liu et al., 2012) or an optic nerve crush model (Vigneswara et al., 2015). It has been shown previously that the PEDF N-terminus (residues 78-121) has the neurotrophic activity of PEDF (Bilak et al., 2002). Becerra's group and others further demonstrated that a 17-mer (corresponding to residues 98-114) contains the PEDF receptor-binding region important for retinal protection (Comitato et al., 2018; Kenealey et al., 2015). Our findings indicate that the 17-mer can partially rescue *Mitf^−/−^* retinal degeneration and so confirm the bioactivity of this fragment. If a similar role can be found in humans, it may open new ways to investigate and treat neurodegenerative diseases associated with PEDF deficiencies.

In sum, our results provide new evidence that MITF can act through neurotrophic factors such as PEDF in RPE cells to maintain retinal function and homeostasis. The findings will not only contribute to our understanding of the underlying mechanisms of retinal degeneration but also provide a means to potentially intervene in retinal degenerations, such as those associated with *Mitf* mutations. We recognize, of course, that the greatly impaired functions of the *Mitf^−/−^* RPE might lead to retinal damage that goes beyond an altered anatomical structure and pattern of gene expression, and that the replenishment of a single trophic factor such as PEDF, or even cocktails of trophic factors, is not sufficient to replace the role of the RPE in regulation of the visual cycle, maintenance of the blood/retina barrier, antioxidant defense or phagocytosis of photoreceptor outer segments. Nevertheless, even a partial rescue of the retina would be of major benefit to patients suffering from degenerative blindness.

## MATERIALS AND METHODS

### Animals

All animal procedures were performed according to a protocol approved by the Animal Care Committee guidelines of Wenzhou Medical University (permit number WZMCOPT-090316). C57BL/6J and ICR WT and *Mitf-vga9* mutant mice were used for this study. For Hematoxylin-Eosin staining, quantification of the ONL thickness was measured from the optic nerve head to the peripheral retina at a distance between 100 μm and 1200 μm and was based on at least six mice in each group. For TUNEL staining and quantification, five mice were used per group. For retina explant experiments, five samples were used per group.

### PEDF peptide and treatment

The PEDF 17-mer (Gln98-Ser115: QRTESIIHRALYYDLIS) peptide was chemically synthesized and purified by China Peptides Co., Ltd. Lyophilized peptide was dissolved in PBS at 1 mg/ml and stored at −80°C. Mice were treated topically with eye drops containing PEDF 17-mer peptide at 1 mg/ml in PBS twice/day from P13 to P20 or from P15 to P20. PBS without peptide served as a control. Eyeballs were collected at P21 for further examinations as described in the Results.

### Retina explant and reconstitution cultures

For retinal explant cultures, P8 WT and *Mitf^−/−^* mice were sacrificed and both eyes were harvested using a curved tweezer. The eyeballs were then transferred to a 35-mm dish with PBS containing Ca^2+^ and Mg^2+^. Intact retina and lens were exposed and isolated by gently tearing the sclera at the optic nerve foramen. For retina explant culture, explants were placed in a drop of medium (100 µl) on a culture nucleopore track-etched polycarbonate membrane (Whatman, 110410) floating in a 35-mm dish filled with 2 ml control culture medium [CM; 45% Dulbecco's modified Eagle medium (DMEM; Invitrogen, 12430), 45% DMEM/F12 (Invitrogen, 11330), 10% fetal bovine serum, 1× Insulin-Transferrin-Selenium (Invitrogen), and 1× HEPES, a modification of the retina explant medium described by Barrasso et al. (2018)] or CM supplemented with PEDF or the 17-mer peptide at different dosages as described in the Results. For recombination experiments, eyeballs from P8 WT and *Mitf^−/−^* mice were harvested and the scleras were torn open as mentioned to obtain the neural retina. From different eyeballs, the corneas were cut and the lens and neural retina extracted to obtain RPE-choroid-sclera complexes. In the latter preparations, RPE cells were mostly attached to the choroid and they were thus used as RPE explants. Neural retinas and RPE-choroid-sclera complexes of the same or different genotypes were recombined in a 100 µl drop of CM on nucleopore track-etched polycarbonate membranes. In these drops, the neural retinal parts at least partially reattached to the RPE-sclera parts. Recombined cultures were maintained for 10 days in CM.

### TUNEL staining

Cryopreserved tissue sections were prepared and TUNEL staining was performed using a TUNEL Kit (Roche, 11 684 795 910), according to the instructions. In brief, frozen sections of mouse retina were permeabilized with 0.1% Triton X-100, 0.1% sodium citrate for 2 min on ice. Then the retina samples were incubated in a TUNEL reaction mixture for 1 h at 37°C. Images were obtained on a Zeiss confocal microscope.

### Immunostaining

For immunostaining, eyes were fixed in 4% paraformaldehyde (PFA) for 2 h, dehydrated in 30% sucrose, and embedded in OCT compound and snap frozen immediately. Sections (12 μm) were collected on a cryostat, dried at room temperature (RT) for 30 min, and fixed in 2% PFA for 10 min. After rinsing, the sections were blocked with 5% bovine serum albumin for 1 h at RT. The samples were incubated overnight at 4°C with specific primary antibodies: anti-cleaved caspase-3 [1:150; Cell Signaling Technology (CST), 9664], anti-rhodopsin (1:200; Millipore, MAB5316), anti-DCT (1:200; Bioworld, BS3320), anti-PEDF (1:100; Abcam, ab180711), anti-OTX2 (1:200; R&D Systems, AF1979) or anti-AIF (1:200; Abcam, ab32516). The staining was revealed by appropriate secondary antibodies [Alexa Fluor^®^ 488 donkey anti-rabbit IgG (H+L) (Life Technologies, A21206), Alexa Fluor^®^ 594 donkey anti-mouse lgG (H+L) (Life Technologies, A21203) and Alexa Fluor^®^ 488 donkey anti-goat IgG (H+L) (Life Technologies, A11055)]. Each staining was performed on slides from at least five animals per condition. Immunostaining results were observed and photographed on a Zeiss confocal microscope.

### Western blotting

Western blotting was carried out as described previously (Ma et al., 2017). Briefly, retinas were lysed to obtain nuclear-enriched and cytoplasm-enriched proteins using a Nuclear and Cytoplasmic Protein Extraction Kit (Beyotime Biotechnology). The purity of the enriched lysates was checked by immunoblotting using the nuclear marker histone H3 (1:1000; CST, 4499T) and the cytoplasmic marker β-actin (1:1000; Santa Cruz Biotechnology, sc-69879).

Equivalent amounts of protein extracts were loaded and separated on 12% SDS-PAGE gels, and then transferred to nitrocellulose membranes (Whatman). The membranes were probed with primary antibodies against AIF (1:1000; CST, 5318T). After incubation at 4°C overnight, the primary antibodies were revealed with the appropriate secondary antibody at RT for 2 h.

### Quantitative real-time PCR

RPE was isolated from WT or *Mitf^−/−^* mice by using the protocol as previously described (Fernandez-Godino et al., 2016). Each group had four samples, and each sample contained the RPE of three mice. Total RNA was extracted from RPE using Trizol reagent (Invitrogen), and reverse transcribed into complementary DNA using random primer and M-MLV reverse transcriptase (Promega). Real-time PCR was performed in triplicate with Power SYBR Green PCR Master Mix on a 7500 Real-Time PCR Detection System (Applied Biosystems). Relative mRNA expression levels were normalized to *Gapdh* and analyzed using the 2^ΔΔCt^ method. Primers used in quantitative PCR are listed in Table S1.

### Statistical analysis

Each experiment was repeated at least four times and results were presented as mean±s.d. All statistical analyses were carried out using SPSS version 20. Student's *t*-test was used for comparisons between two groups and one-way ANOVA was used for comparisons among more than two groups. *P*<0.05 was considered significant.

## Supplementary Material

Supplementary information
