# Supplementary material for: Photoreceptor degeneration in microphthalmia (Mitf) mice: partial rescue by pigment epithelium-derived factor
Source: Dis Model Mech. 2019 Jan 11;12(1):dmm035642. doi: 10.1242/dmm.035642 (PMC6361154; doi:10.1242/dmm.035642)
Supplement: Supplementary information [file dmm-12-035642-s1.pdf]

Supplemental materials

Supplemental Table 1. Real-time PCR primer sequences used in this study

Pedf -Forward	GCCCTGGTGCTACTCCTCT
Pedf -Reverse	CGGATCTCAGGCGGTACAG
Bdnf -Forward	TCATACTTCGGTTGCATGAAGG
Bdnf -Reverse	AGACCTCTCGAACCTGCCC
Ngf -Forward	AGACTCCACTCACCCCGTG
Ngf –Reverse	GGCTGTGGTCTTATCTCCAAC
Pdgf d -Forward	TACAGTTGCACTCCCAGGAAT
Pdgf d –Reverse	CTTCCAGTTGACAGTTCCGCA
Dct-Forward	GTCCTCCACTCTTTTACAGACG
Dct –Reverse	ATTCGGTTGTGACCAATGGGT
Rhodopsin- Forward	CCCTTCTCCAACGTCACAGG
Rhodopsin- Reverse	TGAGGAAGTTGATGGGGAAGC
Opn1sw- Forward	CAGCCTTCATGGGATTTGTCT
Opn1sw- Reverse	CAAAGAGGAAGTATCCGTGACAG
Opn1mw- Forward	ATGGCCCAAAGGCTTACAGG
Opn1mw- Reverse	CCACAAGAATCATCCAGGTGC
Gnat2- Forward	GGATGGCTACTCACCCGAAG
Gnat2- Reverse	TGCATAGTCAATGCCTAGTGTG
Crx- Forward	GTTCAAGAATCGTAGGGCGAA
Crx- Reverse	TGAGATGCCCAAAGGATCTGT
Gapdh- Forward	AGGTCGGTGTGAACGGATTTG
Gapdh - Reverse	TGTAGACCATGTAGTTGAGGTCA

Disease Models & Mechanisms • Supplementary information

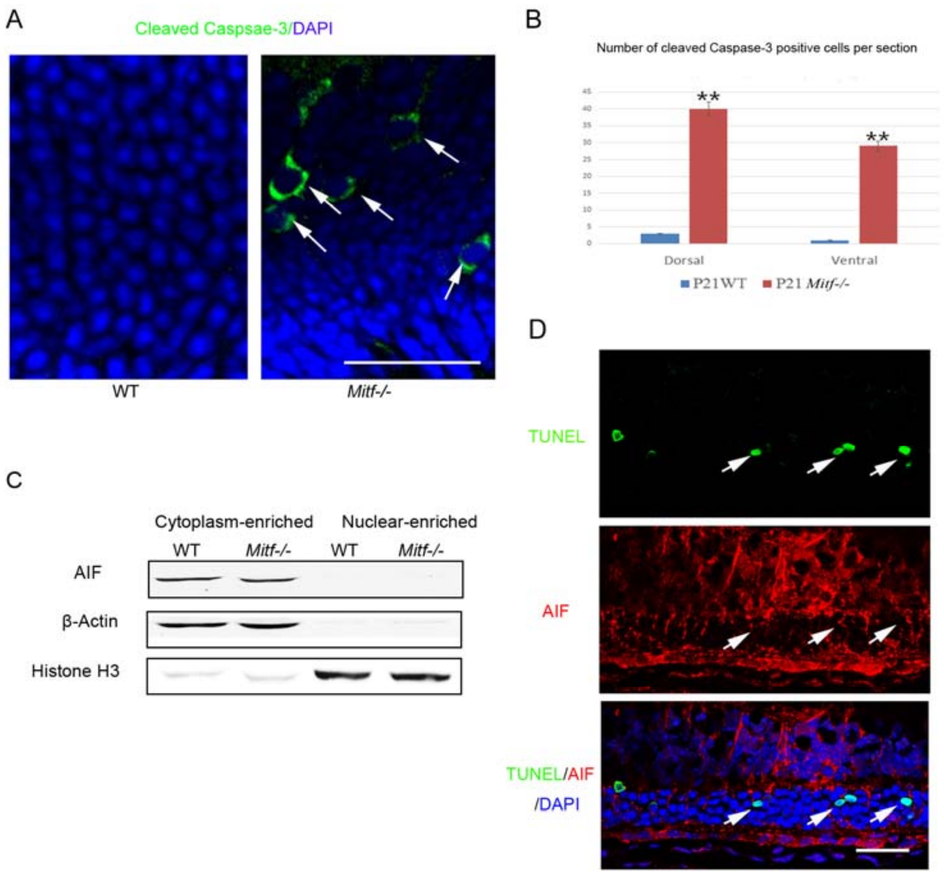

Fig. S1. Apoptosis in *Mitf-/-* retinal degeneration. (A) Representative immunostaining images of P21 WT or *Mitf-/-* ONL for cleaved Caspase-3 (green signals). The white arrows point to cleaved Caspase-3 positive photoreceptor cells. Scale bar, 20 µm. (B) Quantification of the cleaved Caspase-3 positive cells was done based on the results from A. Note that the number of cleaved Caspase-3 positive photoreceptor cells was significantly increased in *Mitf-/-* retina compared to WT. n=5. Results are presented as mean ± SD. ** indicates $P < 0.01$. C. Western blot analysis of AIF in cytoplasm-enriched and nuclear-enriched protein extracts from WT and *Mitf-/-* mouse retinas. The purity of enriched lysates was examined by immunoblotting with anti-actin and anti-Histone H3 antibodies. Note that AIF was not found in nuclear-enriched protein extracts. (D) Representative immunostaining images for AIF (red signals) and TUNEL-positive staining of dying cells (green) in *Mitf-/-* retina. Note that none of TUNEL-positive cells showed nuclear localization of AIF. Scale bar, 20 µm.

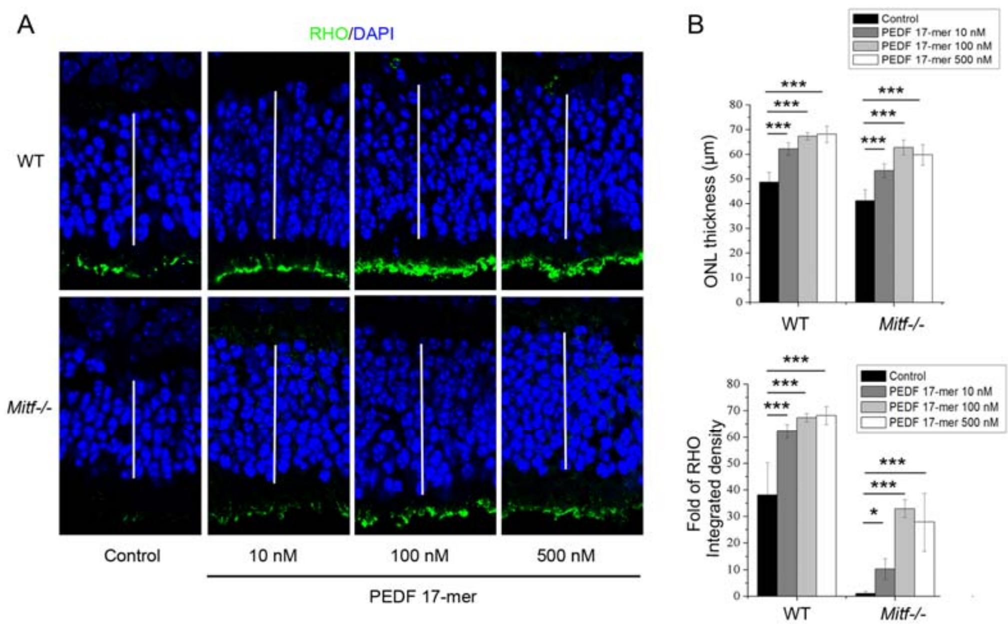

Fig. S2. PEDF 17-mer rescues *Mitf-/-* retinal degeneration in a dose-dependent way in explant cultures. (A) Neural retina explants of WT or *Mitf-/-* mice were prepared and treated with PEDF 17-mer at the indicated doses (n=5). Immunostaining of neural retina explants for RHO was performed. White lines mark the thickness of the ONL. (B) Quantifications of the outer nuclear layer (ONL) thickness and the integrated density of RHO were done based on the results from A. Note that both of ONL thickness and RHO staining in *Mitf-/-* retina were increased with increased PEDF 17-mer dose. Results are presented as mean ± SD. * indicates $P < 0.05$,*** indicates $P < 0.001$.

Disease Models & Mechanisms • Supplementary information
